# Inhibition of Pyruvate Dehydrogenase in the Heart as an Initiating Event in the Development of Diabetic Cardiomyopathy

**DOI:** 10.3390/antiox12030756

**Published:** 2023-03-20

**Authors:** Abdallah Elnwasany, Heba A. Ewida, Pamela A. Szweda, Luke I. Szweda

**Affiliations:** 1 Division of Cardiology, Department of Internal Medicine, University of Texas Southwestern Medical Center, Dallas, TX 75390, USA; 2 Faculty of Pharmacy, Future University in Egypt, Cairo 11435, Egypt

**Keywords:** pyruvate dehydrogenase, diabetes, obesity, metabolic inflexibility, oxidative stress, cardiomyopathy

## Abstract

Obesity affects a growing fraction of the population and is a risk factor for type 2 diabetes and cardiovascular disease. Even in the absence of hypertension and coronary artery disease, type 2 diabetes can result in a heart disease termed diabetic cardiomyopathy. Diminished glucose oxidation, increased reliance on fatty acid oxidation for energy production, and oxidative stress are believed to play causal roles. However, the progression of metabolic changes and mechanisms by which these changes impact the heart have not been established. Cardiac pyruvate dehydrogenase (PDH), the central regulatory site for glucose oxidation, is rapidly inhibited in mice fed high dietary fat, a model of obesity and diabetes. Increased reliance on fatty acid oxidation for energy production, in turn, enhances mitochondrial pro-oxidant production. Inhibition of PDH may therefore initiate metabolic inflexibility and oxidative stress and precipitate diabetic cardiomyopathy. We discuss evidence from the literature that supports a role for PDH inhibition in loss in energy homeostasis and diastolic function in obese and diabetic humans and in rodent models. Finally, seemingly contradictory findings highlight the complexity of the disease and the need to delineate progressive changes in cardiac metabolism, the impact on myocardial structure and function, and the ability to intercede.

## 1. Metabolic Inflexibility and Diabetic Cardiomyopathy

Obesity and type 2 diabetes are independent risk factors for cardiovascular disease and heart failure [1,2,3,4,5]. Even in the absence of hypertension and coronary artery disease, type 2 diabetics often present with abnormalities in the structure and function of the heart, which is clinically defined as diabetic cardiomyopathy [1,2,3]. Systemically, type 2 diabetes is characterized by hyperglycemia and insulin resistance, indicative of defects in the ability to respond to normal changes in fuel availability and regulate glucose utilization in multiple organs [1,2,3]. Despite medical advances in treating systemic metabolic syndrome, about two-thirds of type 2 diabetics die from some form of heart disease. Depending on the stage of type 2 diabetes and the presence of other co-morbidities, including obesity, hypertension, and/or coronary artery disease, cardiomyopathy in type 2 diabetics manifests as diastolic dysfunction that may progress to diminished systolic function, hypertrophic remodeling, and heart failure [1,2,3]. The inability of the heart to appropriately utilize glucose, heavy reliance on fatty acids for energy production (i.e., metabolic inflexibility), and oxidative stress are believed to play causal roles. However, it has proved difficult to map the progression of metabolic changes that occur specifically in the human heart, as diabetic cardiomyopathy normally develops over many years and, until recently, non-invasive in vivo methods for assessment of cardiac metabolism could not distinguish between fuel uptake and oxidation. As such, it is not known how specific changes in cardiac metabolism drive the development of structural/functional abnormalities, and interventions that specifically target metabolic derangements in the heart are lacking. Here we discuss evidence from the literature that inhibition of pyruvate dehydrogenase, the central regulatory site for glucose oxidation [6,7], initiates the development of cardiomyopathy associated with obesity, metabolic syndrome, and type 2 diabetes.

## 2. Regulation of Mitochondrial Fatty Acid and Glucose Oxidation in the Heart

**Scheme 1:**
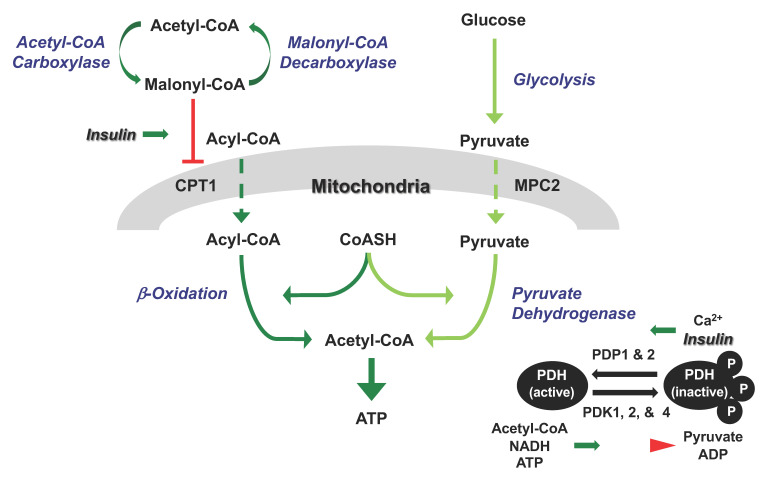
**Mitochondrial metabolic flexibility and regulation in the heart.** The key regulatory sites for fatty acid and glucose-derived pyruvate oxidation within the mitochondria are carnitine palmitoyltransferase 1 (CPT1) and pyruvate dehydrogenase (PDH), respectively. CPT1 transports fatty acids into the mitochondria for fatty acid oxidation (β-oxidation). CPT1 is inhibited by malonyl-CoA. Acetyl-CoA carboxylase (ACC) converts acetyl-CoA to malonyl-CoA, while malonyl-CoA decarboxylase (MCD) decarboxylates malonyl-CoA to form acetyl-CoA. Through unknown mechanisms, insulin increases malonyl-CoA concentrations in the heart, resulting in inhibition of β-oxidation. PDH, which oxidizes pyruvate to acetyl-CoA, is regulated allosterically and by phosphorylation (inactivation) and dephosphorylation (activation). The pyruvate dehydrogenase kinases (PDKs) are activated and PDH is inhibited when fatty acid oxidation produces sufficient acetyl-CoA and NADH for ATP production. Pyruvate dehydrogenase phosphatases (PDPs) are stimulated and PDH is activated by Ca^2+^ in response to beta-adrenergic stimulation and, through unknown mechanisms, by insulin.

The heart adjusts the relative rates of glucose and fatty acid oxidation for energy production depending on available fuel, circulating hormones (insulin) and (ant)agonists, and physiologic demand (e.g., β-adrenergic stimulation) [8] (Scheme 1). Carnitine palmitoyltransferase 1 (CPT1) transports fatty acids into the mitochondria and is a key site for regulation of fatty acid oxidation. Insulin is known to induce acetyl-CoA carboxylase (ACC)-dependent production of malonyl-CoA, an inhibitor of CPT1 and thus β-oxidation [9,10,11,12]. Pyruvate dehydrogenase (PDH) is the central regulatory site for glucose oxidation committing pyruvate to production of the majority of ATP derived from glucose [6,7]. PDH, a mitochondrial enzyme, catalyzes the oxidative decarboxylation of pyruvate to acetyl-CoA and NADH [6,7]. Based on energy demands and glucose and fatty acid availability, PDH is subject to allosteric and posttranslational regulation. Reversible phosphorylation of PDH occurs on three serine residues on the α-chain of the E1 subunit, one of three subunits that comprise the PDH complex. Phosphorylation of PDH inhibits the enzyme and is catalyzed by three isoforms of pyruvate dehydrogenase kinase (PDK1, -2, and -4) in the heart (PDK3 is not expressed in the heart), while dephosphorylation and activation of PDH is catalyzed by two pyruvate dehydrogenase phosphatases (PDP1 and -2) [13,14,15,16,17,18]. When energy demand is low and/or fatty acid oxidation is sufficient, PDKs are activated by ATP, acetyl-CoA, and NADH, resulting in phosphorylation and inhibition of PDH [13,14,15,16,17,18] in a manner that is consistent with the Randle cycle and reciprocal regulation of glucose and fatty acid oxidation [19,20]. In response to β-adrenergic stimulation and increased energetic demand, Ca^2+^-dependent stimulation of PDPs leads to dephosphorylation and activation of PDH [6,7]. Furthermore, it is known that insulin induces dephosphorylation and/or activation of PDH in multiple tissues [21,22,23,24,25,26,27,28,29,30,31]. However, mechanism(s) by which insulin activates PDH in the heart have not been identified. Nevertheless, regulation of PDH and CPT1 are essential components in the control of glucose relative to fatty acid oxidation for energy homeostasis.

## 3. Pyruvate Dehydrogenase Initiates Metabolic Inflexibility in a Model of Obesity/Type 2 Diabetes

Rodents fed high-fat diets are commonly employed experimental models of obesity and type 2 diabetes. As such, a wide body of literature describes the effects of diet-induced changes on systemic metabolism and the structure/function of multiple tissues/processes in rats or mice, with metabolic abnormalities reminiscent of those observed in type 2 diabetic humans [8,32,33,34,35,36,37,38,39,40,41,42,43,44,45,46,47,48,49]. Our published findings indicate that PDH plays a key role in loss of metabolic flexibility in the heart arising from high dietary fat. Within hours of feeding mice a high-fat diet, cardiac mitochondria exhibited an intrinsic decline in the ability to oxidize the glycolytic product pyruvate [35]. This was due to a selective increase in PDK4 expression and subsequent phosphorylation and inhibition of PDH [35]. Thus, in the context of high dietary fat, loss of PDH activity likely initiates metabolic inflexibility and heavy reliance on fatty acid oxidation for energy production (Scheme 1).

## 4. Myocardial Pyruvate Oxidation in Humans with Type 2 Diabetes

Data from humans with type 2 diabetes indicate that diminished pyruvate oxidation precedes reductions in cardiomyocyte glucose uptake, systolic dysfunction, and heart failure. Notably, as judged by noninvasive hyperpolarized ^13^C magnetic resonance imaging (MRI), hearts of type 2 diabetic humans with normal systolic function exhibited diminished pyruvate oxidation and, thus, flux through pyruvate dehydrogenase. This was accompanied by a myocardial energy deficit (phosphocreatine/ATP) and diastolic dysfunction [50]. As such, evidence from diabetic humans [50] and a mouse model of diabetes [35] indicate that inhibition of PDH precipitates metabolic inflexibility prior to the development of diabetic cardiomyopathy.

The use of in vivo hyperpolarized ^13^C MRI represents a significant advance in our ability to measure metabolic changes that initiate metabolic inflexibility. Prior to this technology, the primary means to assess glucose metabolism in vivo was to measure ^18^F-deoxyglucose uptake by positron emission tomography (PET) during euglycemic hyperinsulinemic clamp. It has been reported that type 2 diabetic and non-diabetic patients with preserved ejection fraction or no overt clinical manifestations in the presence or absence of stable coronary artery disease exhibited no difference in the rate of insulin-stimulated myocardial ^18^F-deoxyglucose uptake [51,52,53,54]. In contrast, the rate of ^18^F-deoxyglucose uptake correlated positively with ejection fraction in diabetic patients [55]. Additionally, in heart failure patients with severely depressed ejection fraction (<30%) undergoing coronary artery grafting, ^18^F-deoxyglucose uptake was reduced in type 2 diabetics relative to nondiabetic subjects and in both groups relative to healthy controls [56]. Collectively, these findings suggest that, in humans, type 2 diabetes does not compromise cardiac glucose uptake at early stages of disease when diminished pyruvate oxidation is evident, but rather decreased glucose uptake is observed when clinical manifestations of systolic dysfunction or heart failure are evident. However, to fully understand the role of defects in glucose uptake in the progression of diabetic cardiomyopathy, studies employing ^18^F-deoxglucose require more exhaustive analyses of diastolic and systolic function as well as consideration of patient exclusion criteria, clamp conditions, nutritional state, and time of analysis of subjects. Furthermore, deoxyglucose import is not an accurate measure of glucose/pyruvate oxidation. Glucose phosphorylation by hexokinase rather than transport limits the rate of glucose entry into glycolysis [57], and insulin stimulates glucose oxidation by increasing the activity of cardiac PDH, the rate-limiting step for pyruvate-derived glucose oxidation [21,22,23,24,25,26,27,28,29,30,31].

## 5. Experimental Considerations When Analyzing Pyruvate Dehydrogenase Activity

The contribution of PDH to diet-induced declines in cardiac glucose oxidation and insulin sensitivity may be easily overlooked or concealed based on sample preparation and experimental conditions. The common practice of fasting animals prior to metabolic analyses obscures the impact of diet, as fasting elicits effects on cardiac PDK4 content and PDH activity similar to short-term high dietary fat [35,37,38,58,59,60]. In addition, for assessment of mitochondrial oxidative phosphorylation (e.g., isolated mitochondria or permeabilized muscle fibers), the use of high concentrations of pyruvate (10 mM) in the assay can (re)activate PDH independent of experimental treatment [14,35]. Similarly, analysis of PDH activation state after hearts have been perfused ex vivo may not give an accurate assessment of in vivo changes in enzyme activity, given that perfusate typically contains glucose, fatty acids, and insulin that do not reflect in vivo concentrations, particularly in mice fed high dietary fat. PDK4 is a short-lived protein (<1 h) that is rapidly degraded by the mitochondrial ATP-dependent protease LON under respiratory conditions that promote PDK4 dissociation from the PDH complex [61,62]. Thus, PDK4-dependent PDH inhibition may be underestimated or masked if PDK4 protein is degraded during sample preparation/analysis. Finally, due to time-of-day rhythmicity [63], PDH activity is inherently low when experiments with nocturnal rodents are performed by humans, which could further obfuscate the impact of diet.

## 6. Evidence from Genetic Mouse Models That Diminished Pyruvate Oxidation Can Induce Metabolic Cardiomyopathy

*Deletion of PDHα Subunit*—Embryonic knockout of the alpha subunit of PDH (heart and skeletal muscle) in mice is lethal at postnatal day seven [64]. While the lethal phenotype was overcome by placing PDHα-knockout mice on a high-fat diet, the price of survival was cardiac hypertrophy and diminished systolic function [64]. In a separate study, it was reported that inducible cardiac-specific PDH knockout led to diastolic dysfunction and cardiac hypertrophy [65]. These studies highlight the importance of PDH activity in the maintenance of contractile function.

*Deletion of Mitochondrial Pyruvate Carrier*—In humans [66,67,68] and mouse models [67,68], heart failure is associated with downregulation of the mitochondrial pyruvate carrier (MPC) subunits 1 and 2. MPC transports glucose-derived pyruvate into the mitochondria for oxidation by PDH and for use in biosynthetic pathways. Mice with constitutive or inducible cardiomyocyte-specific knockout of MPC1 or 2 exhibit progressive myocardial hypertrophy, dilation, contractile dysfunction, and failure [66,67,68,69]. In hearts from MPC KO mice, elevated abundance of anabolic metabolites and indices of hypertrophic signaling were observed [66,67,68,69]. In studies that included measurement of cardiac energy status, one group found no change in energy charge [67], while a second observed a drop in ATP level and decreased cardiac glucose oxidation [69]. These observations demonstrate the importance of mitochondrial pyruvate transport and oxidation for maintaining myocardial structure/function. Mechanisms by which loss in MPC induces contractile dysfunction are, however, complex given that certain structural/functional alterations can be rescued by feeding MPC-KO mice a ketogenic or high-fat diet [68,69].

*Transcriptional Regulation of PDK4 Content and PDH Activity*—FOXO1 (forkhead box protein O1) is an insulin-responsive transcription factor important for cell growth and metabolism known to induce upregulation of PDK4, which can phosphorylate and inhibit PDH [70,71]. Mice fed a high-fat diet for an extended duration exhibit hypertrophy, elevated levels of cardiac FOXO1 and *pdk4*, and diminished fractional shortening. Cardiac-specific knockout of FOXO1 prevented high-fat-diet-induced increases in PDK4 transcript levels, loss in systolic function, and hypertrophic growth [72]. In a second model, mice fed a high-fat diet and administered a single dose of streptozotocin exhibited diastolic dysfunction [73]. In this model, cardiac-specific knockout of FOXO1 resulted in diminished *pdk4*, elevated PDH activity, and protection from diastolic dysfunction. Pharmacologic inhibition of FOXO1 was similarly cardioprotective [73]. Beneficial effects of FOXO1 inhibition were not evident in PDH1α-knockout mice [73]. These observations indicate that PDH inhibition contributes to the development of metabolic cardiomyopathy, in part, through FOXO1-mediated upregulation of PDK4.

Collectively, these genetic mouse models (PDHα, MPC, and FOXO1 knockout) indicate that reduced mitochondrial import and/or oxidation of pyruvate are sufficient to induce functional and structural changes to the heart reminiscent of those observed during the development of cardiomyopathy and heart failure in diabetic humans and animal models. Studies with MPC-KO mice also reveal that diminished pyruvate oxidation can increase metabolites to support anabolic processes and hypertrophic growth.

## 7. Potential Mechanisms by Which Metabolic Disruptions Drive the Development of Cardiomyopathy

Mitochondrial pyruvate relative to fatty acid oxidation produces more ATP per mole of oxygen consumed and is therefore an important substrate under high energetic demand [6,7]. In contrast, the rate of oxygen-derived free radical production by the mitochondria is greater with fatty acid relative to pyruvate oxidation. Thus, potential mechanisms by which inhibition of PDH may drive the development of diabetic cardiomyopathy include a reduction in the energetic capacity of the heart, particularly in response to increased physiologic demand, and the induction of a state of chronic oxidative stress.

*Energetic Limitations*—As noted previously, noninvasive in vivo analysis of type 2 diabetic humans with preserved ejection fraction revealed that diastolic dysfunction was associated with reduced flux through PDH and diminished phosphocreatine/ATP [50]. Additionally, in obese humans, the degree of diastolic dysfunction and improvement upon weight loss correlated directly with myocardial phosphocreatine/ATP [74]. In a rodent model of diabetes (high-fat diet and a single dose of streptozotocin) in which PDK4 protein is elevated, diminished pyruvate oxidation in the heart as measured in vivo has been linked to diastolic but not systolic dysfunction [75]. Treatment of diabetic animals with dichloroacetate, a non-specific inhibitor of PDKs, diminished PDK levels and restored pyruvate oxidation and diastolic function [75]. These studies implicate loss in PDH activity and energetic deficits in diastolic dysfunction associated with diabetic cardiomyopathy.

Energetic and functional deficits, however, are not universally observed in rodent models of obesity and accompanying type 2 diabetes. In addition, hearts from PDK4-overexpressing mice, which exhibit severely reduced PDH activity (>90% compared to controls) and an increase in fatty acid and decrease in glucose oxidation, do not exhibit a change in systolic function, although diastolic parameters were not measured [76]. There are several factors that must be considered in order to define the specific impact of PDH inhibition on the structure and function of the heart in the clinical and experimental settings of obesity and diabetes. First, limitations on energy production would most likely initially affect diastolic function. Relative to contraction, a large amount of energy is required to pump Ca^2+^ back into the sarcoplasmic reticulum during relaxation. Diastolic parameters in rodents are not routinely measured and are often evaluated in the anesthetized state, where energy demand is reduced. Second, indices of cardiac energetics and function are generally measured during the day. Thus, analyses are performed during the inactive phase for rodents, when fatty acid oxidation is the predominant source of energy for the heart and would lessen the apparent impact of diminished PDH activity. Third, energy charge (ATP + 1/2ADP/ATP + ADP + AMP) is maintained by reciprocal regulation of energy production and consumption [77]. Phosphocreatine/ATP coupled with diastolic measures may be a more sensitive measure of energetic limitations. Finally, and reflective of the previous considerations, energetic and functional defects arising from inhibition of PDH and diminished glucose oxidation may be most evident when physiologic demand is high. As demonstrated in mice, cardiac hypertrophy and heart failure with preserved ejection fraction (HFpEF) was not induced unless high-fat diet was administered in combination with hypertension upon administration of the nitric oxide synthase inhibitor, L-NAME [78]. Although high-fat diet ± L-NAME resulted in similar increases in PDK4 protein and decreases in PDH activity, the additional stress of hypertension was required to induce diastolic dysfunction, hypertrophy, and HFpEF [79]. Thus, diminished cardiac energetic capacity resulting from high-fat-diet-induced inhibition of PDH may only become evident with increased energy demand.

While it is widely believed that metabolic inflexibility promotes diabetic cardiomyopathy, a recent study demonstrated that enhancing β-oxidation was cardioprotective for mice fed high dietary fat. Acetyl-CoA carboxylase (ACC) produces malonyl-CoA, an inhibitor of CPT1, the mitochondrial fatty acid transporter, and thus inhibits β-oxidation [9,10,11] (Scheme 1). It has been reported that genetically increasing fatty acid oxidation by ACC knockout specifically in the heart protects against high-fat-diet-induced (24 wk) decreases in phosphocreatine/ATP as well as indices of diastolic and systolic dysfunction and hypertrophic growth [80]. Potential mechanisms include preservation of energetic capacity and β-oxidation, consumption of excess fatty acids to prevent accumulation of toxic levels of fatty acids and derivatives (lipotoxicity), and maintenance of Parkin-mediated mitophagy for the removal of damaged mitochondria. The possibility of compensatory changes in metabolism must also be considered. This study highlights the complexity of the pathogenesis of diabetic cardiomyopathy and the potential contributing roles of lipotoxicity, mitochondrial dysfunction, and oxidative stress.

*Metabolically Driven Changes in Mitochondrial Pro-Oxidant Production*—Markers of enhanced oxidative stress have been observed in type 2 diabetic humans with cardiovascular disease and in animal models of diabetic cardiomyopathy [1,2,3,41,49,81]. Indicative of metabolically driven oxidative stress, we have previously demonstrated that high dietary fat induced a rapid increase in the level and activity of the antioxidant enzyme catalase specifically within cardiac mitochondria [82]. Enhanced pro-oxidant production may be caused by increased reliance on mitochondrial fatty acid oxidation necessitated by PDK4-dependant inhibition of PDH [35]. We and others have shown that cardiac mitochondria respiring on fatty acids relative to pyruvate have higher rates of pro-oxidant production [82,83]. A likely mechanism is the manner in which electrons are delivered to the electron transport chain. In contrast to pyruvate oxidation, β-oxidation also delivers reducing equivalents to ubiquinone (CoQ) via the electron transfer flavoprotein downstream of complex I. This results in an increase in CoQH_2_/CoQ and NADH/NAD^+^ enhancing the rate of superoxide anion and ultimately H_2_O_2_ production by the electron transport chain [84,85]. Offering support for increased reliance on β-oxidation as a source of oxidative stress, the level of 8-hydroxyguanosine (8-OHG), a marker of free-radical-mediated DNA damage, increased when mitochondrial fatty acid oxidation was elevated in a cell culture model [86]. Similarly, we have shown that 8-OHG was diminished in hearts from cardiomyocyte-specific PDK4-knockout mice, which exhibited a decrease in fatty acid relative to glucose oxidation [87]. Potentially reflecting changes in fatty acid relative to pyruvate oxidation over the 24-h daily cycle, it has been reported that, in mice, mitochondrial pro-oxidant production in the heart increases during the inactive phase when reliance on β-oxidation is elevated [88].

Increased pro-oxidant production arising from greater reliance on fatty acid oxidation for energy production is unlikely to have an immediate impact on cardiac function. Diurnal changes in pro-oxidant production have been observed [88] and, as we have shown, mice fed a high-fat diet exhibit a rapid antioxidant response in cardiac mitochondria [82]. However, persistent metabolic inflexibility and sustained oxidative stress may lead to an accumulation of damage. Paradoxically, stimulation of fatty acid oxidation appears beneficial in the setting of long-term high dietary fat [80]. Myocardial lipid accumulation has been observed in models of diabetes, leading many to invoke the idea of lipotoxicity in the development of diabetic cardiomyopathy [1,2,3]. Evidence indicates that enhancement of β-oxidation upon ACC knockout prevents the accumulation of lipids while preserving mitophagy and mitochondrial function [80]. Thus, diminished pyruvate oxidation followed by a cascade of events leading to aberrant fatty acid metabolism may lead to mitochondrial dysfunction, amplify oxidative stress, and contribute to diabetic cardiomyopathy and heart failure [1,2,3,49,81].

## 8. Summary and Perspective

Cardiac PDH activity declines rapidly in mice fed a high-fat diet, a model of obesity and type 2 diabetes [35]. Inhibition of PDH necessitates increased reliance on fatty acids, which enhances mitochondrial pro-oxidant production [82,83]. Insulin resistance, metabolic inflexibility, and oxidative stress, hallmarks of type 2 diabetes [1,2,3], can therefore be initiated by a high-dietary-fat-induced inhibition of PDH. In vivo analysis of diabetic humans supports a role for diminished PDH activity in declines in cardiac energy homeostasis and diastolic function [50].

As discussed, changes in the activity of PDH in the context of diabetes and cardiomyopathy are easily overlooked. The activation state of PDH may not be preserved upon ex vivo and in vitro analyses and can be masked by methods of sample preparation. Further, PDH activity is highly susceptible to physiologic (β-adrenergic) and nutritional stress and exhibits diurnal rhythmicity [63]. Thus, results will be greatly influenced by the time of day, nutritional state, and anxiety of the organism when analyses are performed. Perhaps most strikingly, it has been known for decades that insulin inhibits fatty acid oxidation while stimulating glucose-derived pyruvate oxidation [8,9,10,11,12,21,22,23,24,25,26,27,28,29,30,31]. Given the importance of this regulation for cardiac metabolism and disease, it is alarming that the mechanism(s) by which insulin stimulates PDH and inhibits β-oxidation in the heart have not been fully elucidated and have often been inferred from other organs that are metabolically distinct from the heart (Scheme 1). Insulin is known to induce the production of malonyl-CoA, an inhibitor of the mitochondrial fatty acid transporter CPT-1A [9,10,11,12]. It is not known how this process is regulated in the heart. Mechanisms could include allosteric, posttranslational, or substrate-level regulation of acetyl-CoA carboxylase and/or malonyl-CoA decarboxylase, enzymes that produce and consume malonyl-CoA, respectively (Scheme 1). Likewise, insulin is known to stimulate dephosphorylation and activation of PDH in multiple tissues [21,22,23,24,25,26,27,28,29,30,31]. While insulin-dependent activation of PDH in the heart appears to require AKT phosphorylation [25], the mechanism remains elusive. Fundamental mechanisms by which insulin regulates cardiac metabolism must be delineated if disruptions in these processes are to be fully understood and interventions developed relevant to diabatic cardiomyopathy.

Studies utilizing mouse models of obesity, diabetes, and heart failure must consider strain-specific differences and validate results in multiple models. For example, C57BL/6N and C57BL/6J mice are commonly used strains in the field. However, C57BL/6J substrains bear mutation(s)/deletion(s) in the gene for nicotinamide nucleotide transhydrogenase (NNT), an enzyme involved in NAD(P)H/NAD(P)+ homeostasis and, thus, redox regulation, and they have other genotypic differences [89]. It has been reported that these strains exhibit differences in systemic metabolism, mitochondrial H_2_O_2_ production, obesity, and the development of diabetes [36,90,91,92]. To our knowledge, no studies directly comparing the effects of high-fat diet on myocardial metabolism and the development of cardiomyopathy and heart failure in C57BL/6J and C57BL/6N mice have been performed.

While diabetic cardiomyopathy is likely multifactorial, mechanisms thought to contribute are often inferred from studies conducted in failing hearts. Metabolic inflexibility, mitochondrial dysfunction, lipotoxicity, oxidative stress, aberrant hypertrophic signaling, and inflammation have all been implicated in structural and functional changes associated with diabetic cardiomyopathy and heart failure [1,2,3,49,80,81,82]. However, it is not known how early metabolic alterations drive the progression from diastolic dysfunction to hypertrophy and/or heart failure. Multiple interrelated pathways are likely to be affected during disease pathogenesis. It will be important to determine whether mechanisms underlying diastolic and systolic dysfunction are distinct or involve a continuum of events perpetuated by early metabolic adaptations. What is necessary is that the progression of the disease be carefully tracked in diabetic humans and animal models to provide a clearer mechanistic understanding of the causal relationships between specific events and the timeframe in which they occur. Recent advances in hyperpolarized ^13^C magnetic resonance imaging [50] and stable isotope labeling/tracing [93] allow in vivo assessment of glucose oxidation and the fate of glucose-derived carbons. These techniques can be used to measure the progression of metabolic changes along with in vivo measures of cardiac structure, function, and energy status, thus facilitating the development of interventions to identify and target underlying causes at appropriate stages of disease. Evidence indicates that the pathogenesis of diabetic cardiomyopathy is complex, requiring careful consideration of the etiology of progressive alterations in metabolism and redox status in multiple models relevant to the human condition.

PDH is an attractive target for metabolic intervention early in the development of diabetic cardiomyopathy. However, promotion of fatty acid oxidation in a genetic mouse model (ACC KO) has also proven beneficial in the setting of high-dietary-fat-induced cardiomyopathy [80]. Thus, to prevent and/or treat diabetic cardiomyopathy in humans, a more efficacious strategy might be to seek metabolic interventions that promote a return to metabolic flexibility rather than solely targeting PDH or aspects of fatty acid oxidation. This will require a deeper mechanistic understanding of the regulation of metabolic flexibility in the heart. For example, much of what is known of inhibition of PDH in diabetes involves high-fat-diet-induced increases in PDK4. A closer look at other kinases (PDK1 and PDK2), phosphatases (PDP1 and PDP2), and what controls their association with the PDH complex and degradation may shed light on targets for intervention. Furthermore, the impact of known treatments for diabetes (i.e., insulin and metformin) on specific components of pyruvate and fatty acid oxidation must be defined in an effort to identify new therapeutic strategies. Finally, we have reviewed literature on the benefits of knockout of the transcription factor FOXO1 in preventing high-fat-induced cardiomyopathy [72,73]. Other transcription factors that influence fatty acid and glucose oxidation should be considered. For example, PGC-1α, PPARα, and ERRα are responsive to nutritional status and the time of day and impact the content of enzymes involved in β-oxidation that can, in turn, influence PDK4 levels and PDH activity [8,37,59,94,95]. Overall, the progression of numerous metabolic changes, molecular mechanisms by which they occur, the interrelationships between these events, and the effects on the structure and function of the heart must be more rigorously delineated if effective metabolic interventions to prevent and/or treat diabetic cardiomyopathy are to be established.

## Data Availability

All data are contained within this article.
